# How to Assess the Epistemic Wrongness of Sponsorship Bias? The Case of Manufactured Certainty

**DOI:** 10.3389/frma.2021.599909

**Published:** 2021-05-05

**Authors:** Jon Leefmann

**Affiliations:** Center for Applied Philosophy of Science and Key Qualifications (ZiWiS), Friedrich-Alexander-Universität Erlangen-Nürnberg, Erlangen, Germany

**Keywords:** sponsorship bias, manufactured certainty, epistemic wrongness, error, social epistemology, evidence, confirmation

## Abstract

Although the impact of so-called “sponsorship bias” has been the subject of increased attention in the philosophy of science, what exactly constitutes its *epistemic* wrongness is still debated. In this paper, I will argue that neither evidential accounts nor social–epistemological accounts can fully account for the epistemic wrongness of sponsorship bias, but there are good reasons to prefer social–epistemological to evidential accounts. I will defend this claim by examining how both accounts deal with a paradigm case from medical epistemology, recently discussed in a paper by Bennett Holman. I will argue that evidential accounts cannot adequately capture cases of sponsorship bias that involve the manufacturing of certainty because of their neutrality with respect to the role of non-epistemic values in scientific practice. If my argument holds, it further highlights the importance of integrating social and ethical concerns into epistemological analysis, especially in applied contexts. One can only properly grasp sponsorship bias as an epistemological problem if one resists the methodological tendency to analyze social, ethical, and epistemological issues in isolation from each other.

## Sponsorship Bias As an Epistemic Phenomenon

In recent years, sponsorship bias has been widely discussed in relation to bias in science (Holman and Bruner, 2015; Holman and Elliott, 2018). The term refers to the fact that research funded by industries or other commercial enterprises is more likely than publicly funded research to produce results in line with the funder's commercial interests (Lexchin et al., 2003; Sismondo, 2008; Lundh et al., 2017). Hence, it is also sometimes called preference bias (Wilholt, 2009). There is, however, disagreement about how to best explain this phenomenon. First, there is a debate about whether the phenomenon is primarily a form of bias (Wilholt, 2009; Holman and Bruner, 2017; Holman and Elliott, 2018; Robinson, 2019; Reutlinger, 2020b), *viz*., an epistemic shortcoming, or whether it should instead be interpreted as an ethical or political problem (Melo-Martín, 2019). Second, as we will see below, epistemic analyses of sponsorship bias differ over how to explain its epistemic wrongness.^1^

An epistemic analysis of sponsorship bias can be supported by noting the various mechanisms that enable incorrect conclusions to be drawn from scientific data. For example, the preference for one study design over another is known as design bias. Other examples concern forms of data-selection bias (favoring certain data when presenting research results), interpretation bias (favoring one interpretation and disregarding alternatives), and publication bias (only publishing results that confirm a preferred hypothesis while holding back or even suppressing results that do not).^2^ These distorting mechanisms can function at the level of an individual researcher and of an entire scientific community. For example, an individual researcher can disregard certain data when drawing conclusions from an experiment, while a research group can follow rules and practices that promote flawed data analysis, false conclusions, and erroneous interpretations. These mechanisms always result in epistemic shortcomings, insofar as they cause researchers to adopt insufficiently supported or even false beliefs. Like any account of bias, an analysis of sponsorship bias must explain exactly what goes epistemically wrong in all such cases.

However, the role of preference in these biases shows that they are not merely epistemic. If sponsorship bias resulted from flawed reasoning and logical mistakes alone, it would be better described as an error, rather than a bias. But *preferring* one set of data over another, or *deciding* not to publish uncharitable research, are forms of practical reasoning. This suggests that sponsorship bias does not involve mere epistemic wrongness but, rather, such wrongness that is consciously or unconsciously motivated by practical interests or preferences. A full account of sponsorship bias must, therefore, also explain the role of practical interests in bringing about epistemic wrongness.^3^

The most obvious response to this challenge is to simply insist that the epistemic wrongness of biased research stems from the influence of political or financial interests on the research process. Science is thus imagined to be a purely epistemic endeavor^4^, which is then tainted by concerns that compromise the pure pursuit of knowledge by motivating scientists to produce results that are socially acceptable, politically desirable, or supportive of social change.

Current mainstream philosophy of science would, however, not welcome this answer. The idea that science can be totally free of non-epistemic values has long been recognized as a philosophical ideal that cannot be realized in practice. To insist that only research wholly free of social, political, or practical values and interests is epistemically apt would be to repudiate the epistemic credentials of almost all actual science. In recent decades, various philosophers have argued that social, political, and practical values play a role in science, not only in relation to the choice of research agendas but also within the research process (Rudner, 1953; Longino, 1990; Douglas, 2000, 2007). The argument from inductive risk, for example, purports to show that scientists inevitably decide whether to accept or reject a hypothesis in light of evidence about the relative harmfulness of either endorsing a hypothesis that is, in fact, false, or rejecting one that is, in fact, true (Rudner, 1953; Hempel, 1965; Douglas, 2009). The harmfulness of making these mistakes cannot be evaluated without reference to practical, social, or political—i.e., non-epistemic—values.^5^

If we side with the current mainstream in the philosophy of science and accept that science is inherently value laden^6^, we cannot account for the epistemic wrongness of biases (and of sponsorship bias, in particular) by simply pointing to the non-epistemic interests and preferences of those involved. Epistemically unimpeachable research would also be influenced by such values. One could, of course, point out that there is no problem with non-epistemic values as such, but only when certain such values are involved, such as purely commercial concerns to maximize financial returns on research. Even setting aside cases where the intrusion of such concerns into science does not cause epistemic problems (Carrier, 2008), this still raises the question on how to distinguish between acceptable and unacceptable non-epistemic values, and this distinction would have to be justified by reference to pragmatic or ethical rather than epistemic principles. It is logically impossible to justify the validity of *non-epistemic* values using *epistemic* criteria.^7^

Philosophers have offered various explanations of the highly plausible intuition that sponsorship bias is at least as much of an epistemic problem as an ethical one and of what exactly goes epistemically wrong in cases of sponsorship bias. Borrowing terminology from Reutlinger (2020a), one can divide these into “evidential accounts” (EAs) and “social epistemological accounts” (SEAs). In what follows, I will argue that SEAs are better suited than EAs to account for important features of sponsorship bias. I will defend this claim by discussing the two types of accounts through the lens of a paradigmatic example used in discussions of evidence hierarchies in medical epistemology: the anti-arrhythmic drug case (AAC). This example was recently offered by Bennett Holman as a case of sponsorship bias (Holman, 2019).

The paper proceeds as follows: *The Anti-Arrhythmic Drug Case* section and the *Sponsorship Bias as Manufactured Certainty* section introduce Holman's interpretation of the AAC as a paradigm case of manufactured certainty. *The Evidential Conception of Epistemic Wrongness* section and *The Social Epistemological Conception of Epistemic Wrongness* section briefly discuss, respectively, Reutlinger's evidential account of epistemic wrongness and Wilholt's social epistemological account. The *Challenging the Evidential Account* section, *The Problem of the Target Level* section, and the *Challenging the Social Epistemological Account section* analyze how these accounts deal with cases of manufactured certainty. In these sections, I will also argue that EAs fail to explain the AAC as an instance of manufactured certainty, while SEAs succeed in doing so, at least on the level of building expert consensus. I conclude that the social epistemological account should be preferred over the evidential account based on its higher explanatory potential in cases like this.

## The Anti-Arrhythmic Drug Case

In order to evaluate the two analyses of sponsorship bias, I shall utilize a socially contextualized version of a paradigmatic case study that is typically interpreted to show the superiority of statistical evidence over mechanistic evidence in clinical decision making (Howick, 2011). Holman takes the socio-political context of the standard version of this case study into account and argues that it presents an instance of massive sponsorship bias. He concludes that the case does not provide sufficient grounds to favor statistical over mechanistic evidence (Holman, 2019) and that the framework of social epistemology is much more useful than that of traditional epistemology for analyzing collective epistemic practices in medicine (Holman, 2019). I will illustrate these points by presenting both versions of the case, but will focus my attention on the contextually enriched version that highlights the role of the pharmaceutical industry's financial interests. Howick presents the standard version as follows:

Myocardial infarction often damages the muscle and electrical system in the heart, leaving it susceptible to arrhythmias. A common type of arrhythmia, ventricular extra beats (VEBs), occurs when the left ventricle contracts before it has had time to fill completely. The heart then fails to pump sufficient blood. Without treatment, lung, brain, and kidney damage ensues. Worse, VEBs can also degenerate into ventricular fibrillation, or complete electrical chaos. Sudden death soon follows ventricular fibrillation in the absence of electric shock. Large-scale epidemiological studies suggested that between 25 and 50% of sudden cardiac deaths were associated with arrhythmias […]. Based on this understanding of the underlying mechanisms, several drugs were developed and found to be successful for regulating VEBs […]. The drugs became widely prescribed in the belief that they would reduce cardiac deaths (Howick, 2011, p. 126).

A […] comparative clinical study […] the Cardiac Arrhythmia Suppression Trial (CAST), which began in 1987, […] was designed to test whether antiarrhythmic drugs would reduce mortality in patients who had suffered from myocardial infarction (heart attack). In the study, 27 clinical centres randomized (sic!) 1,455 patients to receive encainide, flecainide, or placebo, while 272 were randomized to receive moricizine or placebo. In April 1989 the encainide, flecainide, placebo arm of the study was discontinued because of excess mortality in the experimental groups; 33 of 730 patients (4.5%) taking either encainide or flecainide had died after an average of 10 months follow-up, while only nine of 725 patients (1.2%) taking placebo had died from arrhythmia and non-fatal cardiac arrest over the same time period. The experimental drugs also accounted for higher total mortality (56 of 730, or 7.7% vs. 22 of 725 or 3.0%). Similar negative results were soon found for moricizine (Howick, 2011, p. 124).

Howick presents this case in order to argue that relying on mechanistic evidence for clinical decision making can have fatal consequences when the underlying physiological mechanisms are complex and insufficiently understood. He argues that the case shows that mechanistic evidence is not only unnecessary to establish causal relations but also that basing one's judgments on statistical evidence from randomized clinical trials does a better job in many cases (Howick, 2011). Holman argues, however, that the standard version omits the broader context concerning how and why the medical profession first decided to rely on mechanistic evidence. He contends that the decision to rely on mechanistic evidence was made despite considerable disagreement among medical experts. Holman's version of the case focuses on the definition of the clinical endpoints of the studies that were needed by the pharmaceutical companies to gain FDA approval for their drugs and on the role of the pharmaceutical industry in establishing these endpoints. His reconstruction adds three important points that cast the incident in a completely different light and explain how belief in mechanistic evidence became prominent in the cardiology community in the first place.

First, Holman notes that even highly accredited experts who promoted the hypothesis that VEBs precipitate sudden cardiac death, such as Bernard Lown, warned that VEBs needed to be suppressed “in only a minority of patients, who usually have ischemic heart disease and a life-threatening or symptomatically disabling arrhythmia” (Lown, 1979, p. 321). This shows that there was actually a scope for interpretation about which therapeutic interventions would be justified if, as hypothesized, suppressing VEBs could help to prevent cardiac arrest. At least from Lown's widely respected perspective, the truth of hypothesis would not have licensed the widespread prescription of the anti-arrhythmic drugs.

Second, Holman explains that the FDA and the pharmaceutical companies together organized a conference to determine what kind of evidence concerning the drugs' efficacy would be required for its approval for therapeutic purposes. The aim was to achieve expert consensus as to whether the clinical trials preceding approval should use death as the endpoint of the study or whether a surrogate endpoint such as the suppression of VEBs would suffice. The expert panel led by cardiologist Joel Morganroth consisted of various academic researchers, industry representatives, and members of the FDA cardio-renal division. Morganroth received support from various pharmaceutical companies to determine the agenda of conference and to frame the subsequent discussions. Holman reports that the speakers at the conference were primarily proponents of industry-friendly positions and favored VEB suppression as an adequate (and cost-efficient) endpoint for the studies. He reports, furthermore, that Morganroth actively used his position to prevent critical discussions of the VEB suppression hypothesis when these were demanded several times by critical researchers during the conference. Even though it was obvious that there was considerable disagreement among the experts in attendance about the therapeutic role of VEB suppression, Morganroth was able to build a strong coalition in favor of the surrogate endpoint. The FDA ultimately accepted this conclusion, even though several FDA members explicitly acknowledged that VEB suppression was not enough to guarantee the therapeutic effectiveness of the drugs. The conference not only reached a decision about the endpoint of the clinical studies but also gave the impression that the relevant experts all endorsed the VEB suppression hypothesis (Holman, 2019).

Third, after approval of the endpoints for clinical trials, several pharmaceutical companies launched a marketing campaign for their upcoming drugs. This campaign included efforts to increase the number of industry-friendly scientific publications on this topic by publishing the same study multiple times in several high-ranking medical journals and, in some cases, hindering the publication of contrary evidence. This campaign was complemented by increased funding for researchers, such as Morganroth, who promoted the VEB suppression hypothesis. Several pharmaceutical companies also distributed copies and reprints of favorable studies to doctors to raise awareness of their upcoming products and hired industry-friendly researchers to conduct cardiology seminars for doctors who might later prescribe the drugs. They also engaged selected cardiologists in so-called seeding trials, allowing them to acquire experience of the drugs before they went to market and to compare them to competing treatments (Holman, 2019).

I will assume, for the purposes of this paper, that Holman's enriched version of the anti-arrhythmic drug case is correct. Holman's version not only undermines Howick's interpretation of the case as revealing the insufficiency of mechanistic evidence but also presents it as a case of massive sponsorship bias.^8^ I will argue that it also poses new challenges for the two kinds of accounts of the epistemic wrongness of sponsorship bias. First, it challenges evidential accounts because it shows that decisions about study endpoints and about the kind of evidence necessary to support a hypothesis cannot be explained by reference to confirmation theory. False claims about evidential confirmation relationships can only constitute epistemic wrongs relative to some predefined standard. Second, it challenges social epistemological accounts because it shows that compliance with the methodological standards of a scientific community can have epistemically detrimental results. I will argue, however, that social epistemological accounts can respond to this challenge, while evidential accounts cannot.

My argument will proceed as follows: I will first show that Holman's enriched version of the case represents an instance of sponsorship bias. This will involve identifying the instances of the research that contributed to the anti-arrhythmic drug disaster were actually affected by sponsorship bias. Second, I will explain the challenge to EAs in more detail and show why they cannot fully account for the features that make the example a case of sponsorship bias. Finally, I will explain how this case poses a challenge to SEAs because it shows that infringement of methodological standards is irrelevant to the ascription of epistemic wrongness.

## Sponsorship Bias As Manufactured Certainty

Holman's enriched version of the case constitutes a *prima facie* drastic case of sponsorship bias. However, because many of the practices described in the case might equally shape research that produces valid results, it is necessary to ask whether AAC is a representative case. As I will show, AAC instantiates a range of strategies that is widely used by the pharmaceutical industry. These strategies promote epistemic errors by leading to the adoption of inappropriate research designs. The enriched version of AAC also permits an interpretation that contains two important criteria for sponsorship bias, namely, the occurrence of an epistemic wrong and the generation of this wrong by some kind of practical interest.

A plausible interpretation of AAC would be that the epistemic wrong consists in a research design that is adequate for determining whether the drugs suppress VEBs but that is inadequate to determining whether the drugs have any therapeutic effect. Hence, claiming that the drugs had a therapeutic effect—a claim that was made by researchers in several publications and disseminated by the marketing campaign—was epistemically unjustified, as this had not been shown by the studies that used VEB suppression as an endpoint. This epistemic error was only identified in the subsequent comparative clinical study.^9^

This epistemic wrong was clearly facilitated by practical interests. This is revealed by the influence of the pharmaceutical industry in shaping the make-up and conclusions of the expert panel and the subsequent marketing campaign, which helped create the impression that VEB suppression was accepted by the relevant experts as a guarantee of therapeutic success. The acceptance of the VEB suppression hypothesis, which led to the anti-arrhythmic drug disaster, thus stands as a clear case of manufactured certainty, that is, the impression of certainty over issues that are actually contested.

It is remarkable, furthermore, that the AAC involved several well-known strategies that powerful industries typically use to promote their products. Most of these strategies were pioneered by the tobacco industry from the 1950s onward and are often referred to as the tobacco strategy (Oreskes and Conway, 2010), though they have since been copied by several other industries. The tobacco strategy seeks to hinder the production of scientific knowledge contrary to the interest of the industry. The strategy has five elements: an emphasis on scientific uncertainty, the support of friendly research, the recruitment of distinguished scientists, the creation of an echo chamber effect, and attacks on unfavorable scientific research (Fernandez Pinto, 2017). The pharmaceutical industry did not utilize all of these strategies in AAC, and those it did take up were pursued in a comparatively less aggressive way than by other industries.^10^ In AAC, the industry concentrated on recruiting distinguished researchers (so-called key opinion leaders, such as Morganroth) who promoted their position, gaining the support of friendly research, and on creating an echo chamber effect through their marketing efforts, to get their message across to the medical community. On the other hand, attacks on unfavorable research, if they occurred at all, seem to have been rather indirect, such as refusing to fund critical research. Unlike other uses of the tobacco strategy, the pharmaceutical industry did not wish to manufacture doubt or uncertainty in this case. As the description of the expert panel shows, the pharmaceutical companies rather aimed at *promoting certainty* over an issue (the VEB suppression hypothesis) that was actually uncertain and heavily contested within the research community. In sum, these efforts served to distort the academic discourse on the therapeutic efficacy of the anti-arrhythmic drugs, such that the industry-friendly position gained higher visibility than dissenting views in scientific publications and the medical community.

These observations clearly confirm that AAC can be read as a case of sponsorship bias. Let us now consider how this case challenges evidential and social epistemological accounts of this bias.

I will first briefly survey the distinctive features of these two groups of accounts by examining paradigmatic formulations of each: Reutlinger's evidential account of epistemic wrongness (Reutlinger, 2020b) and Wilholt's social epistemological account (Wilholt, 2009, 2013).

## The Evidential Conception of Epistemic Wrongness

Reutlinger defends an evidential account of epistemic wrongness, according to which “research affected by sponsorship bias is epistemically wrong if and only if the researchers in question make false claims about the (degree of) evidential support of some hypothesis H by data E” (Reutlinger, 2020b).

This statement primarily concerns the nature of epistemic wrongness in the empirical sciences. A scientific claim is wrong insofar as it is not sufficiently supported by evidence. This account of epistemic wrongness is introduced as the defining epistemic property of sponsorship bias, so Reutlinger's formulation seems to imply that there could, in principle, be cases of sponsorship bias in which researchers only make claims that are sufficiently supported by the evidence and that would not therefore constitute cases of epistemic wrongness. This implication seems conceptually disturbing—can research be affected by bias but nonetheless be epistemically flawless?—but I will not concern myself with this problem here. Rather, I will take for granted that biased research by definition contains an element of epistemic wrongness and that this holds *ipso facto* for research affected by sponsorship bias.

Reutlinger defends this evidential account by applying insights from confirmation theory to paradigmatic cases of sponsorship bias, such as the Bisphenol A case (vom Saal and Hughes, 2005; Wilholt, 2009; Carrier, 2013; Biddle and Leuschner, 2015), the Celebrex Case (Brown, 2008), and the tobacco strategy (Oreskes and Conway, 2010; Proctor, 2012). According to Reutlinger, the epistemic error in these different cases can be explained by reference to epistemic principles derived from Bayesian confirmation theory (Earman, 1992; Sprenger and Hartmann, 2019) and frequentist hypothesis testing (Mayo, 2011a,b). These theories of evidential confirmation explain what it means for a set of data E to provide evidential support for a hypothesis H and thereby formulate accounts of what it means to be epistemically justified in believing H in light of the available evidence. It is important for evidential accounts of sponsorship bias to invoke such principles of epistemic justification because such accounts explain the epistemic wrongness of a belief in terms of a lack of epistemic justification for holding the belief as true.

The empirical sciences typically conceive of epistemic justification in terms of evidential confirmation.^11^ Bayesian confirmation theory and frequentist hypothesis testing are currently the most widely accepted theories of evidential confirmation (Reutlinger, 2020b). Both are probabilistic theories. According to the Bayesian confirmation theory, evidence E supports a hypothesis H, if and only if the probability that H is true given E and relevant background knowledge K is higher than the probability that H is true given only the relevant background knowledge K, or more formally: P(H|E, K) > P(H|K). Applications of this Bayesian principle, however, require consideration of a further principle, that of complete local evidence. This latter principle states that one ought to always consider all *available* data produced in an experiment or series of experiments whenever one wishes to establish the degree of confirmation of a hypothesis. The principle of complete local evidence ensures that Bayesian assessments of subjective probabilities take into consideration potentially defeating evidence and so guards against confirming hypotheses based on selective data.

Reutlinger claims that one or both of these basic epistemic principles are typically violated in paradigmatic cases of sponsorship bias. For example, the famous Bisphenol A case (vom Saal and Hughes, 2005) can be interpreted as a case of biased research because the researchers made false claims about the evidential support for their hypothesis that low doses of Bisphenol A do not increase cancer rates in laboratory rats of the CD(SD) strain. The researchers violated the principle of complete local evidence because there was evidence available at the time that CD(SD) rats are insensitive to estrogens and that Bisphenol A functions as an endocrine disruptor and hence strongly influences the effects of estrogens. This defeating evidence was not taken into consideration. Consequently, the researchers also violated the epistemic principle. By claiming that low doses of Bisphenol A do not increase cancer rates in laboratory rats of the CD(SD) strain, researchers suppressed relevant background knowledge K (i.e., CD(SD) rats are insensitive to the effects of low doses of Bisphenol A) but nevertheless claimed that the results of their experiments supported their hypothesis, or more formally: P(H|E) > P(H|E, K). This is the exact opposite of what Bayesian confirmation theory demands.

Reutlinger suggests that the other two cases can be interpreted similarly. In the Celebrex case (Brown, 2008), researchers violated the principle of complete local evidence because they based their claim that the anti-arthritis drug Celebrex caused fewer side effects than its competitors on evidence from only the first 6 months of their study. Had they considered all available evidences from their own research, the study would not have supported this claim. Focusing on partial evidence instead of complete local evidence ignores available and potentially defeating evidence.

In the context of the tobacco strategy, Reutlinger introduces the case of a researcher who claimed in court that smoking cannot be said to cause lung cancer because being a cause in a scientific sense requires constituting a *necessary and sufficient condition* for an effect.^12^ This however, is clearly not the case, as there are people who smoke and never get lung cancer, as well as people who get lung cancer despite never having smoked. In terms of Bayesian confirmation theory, the researcher did not violate either of the two principles in making this claim, but instead confused the very idea of evidential confirmation from which these principles derive. Evidential confirmation operates in probabilistic terms, that is, a hypothesis is more or less likely to be true depending on the degree of confirmation derived from the available evidence. That empirical evidence alone can never establish the necessary and sufficient conditions of an effect has been recognized at least since David Hume's discussion of causation (Hume, 1748/2009).

Reutlinger's evidential account thus explains paradigmatic cases of sponsorship bias as cases in which scientists make false claims based on a misconception of evidential support relationships. On this account, the epistemic wrongness of sponsorship bias is, therefore, primarily a feature of the scientist's assertions and not of their epistemic practices. The researchers in the above cases made false claims insofar as they were unjustified in making these claims given the evidence that was actually available to them. The problem in the Bisphenol A case, for example, was not that the researchers used the insensitive CD(SD) rat strain but that they could have known (and, indeed, probably knew) that using this strain in an experiment could not provide evidence that could actually confirm their hypothesis and yet nevertheless claimed that it did. This shows that evidential accounts tend to construe biased research as analogous to erroneous research. Errors occur due to deviations from valid and generally accepted epistemic principles and researchers can be blamed for committing such an error if they knew or should have known the relevant principles.^13^ Such errors must, however, be distinguished from false beliefs that do not originate from such epistemic deviations and have no implications for blameworthiness. EAs show that bias and error are similar insofar as bias not only indicates a false belief but a false belief that should (and often could) have been avoided. Biased research and erroneous research are thus epistemically wrong for the same kinds of normative reasons.^14^

## The Social Epistemological Conception of Epistemic Wrongness

Social–epistemological accounts (SEAs) of the epistemic wrongness of sponsorship bias approach the phenomenon from a different angle. Like EAs, they share the intuition that there is something genuinely epistemically wrong in cases of sponsorship bias, but they explain the relevant epistemic shortcomings in terms of epistemic social practices rather than evidential support. This has the advantage of better accounting for the social mechanisms that lead to epistemic wrongs in a specific research setting.

I will here treat Wilholt's widely discussed SEA as paradigmatic of this type of account (Wilholt, 2009, 2013). Wilholt argues that the epistemic wrong in cases like those discussed above consists in “the infringement of an explicit or implicit conventional standard of the respective research community in order to increase the likelihood of arriving at a preferred result” (Wilholt, 2009, p.99). He argues in a more recent work that such conventional methodological standards are epistemically relevant on a collective level because they enable mutual trust between the members of a research community and so help to coordinate the joint activity of scientific knowledge production (Wilholt, 2013, 2016). An important motivation for SEAs, and for Wilholt's account in particular, is the critique of the value-free ideal of science. If one takes seriously the insight, mentioned above, that all empirical research involves making judgments based on non-epistemic values and that complete freedom from such values cannot be the hallmark of unbiased research, it seems impossible that accepting the truth of a hypothesis could be epistemically justified solely on evidential grounds. As the argument from inductive risk shows, value judgments about the consequences of falsely accepting a hypothesis are necessarily invoked when determining the degree of evidential confirmation necessary to endorse a hypothesis. If the stakes are high, and the consequences of false acceptance are sufficiently bad, a higher degree of confirmation will be necessary than in cases where less is at stake. Wilholt argues that it is impossible to objectively determine the degree of confirmation necessary for accepting a hypothesis and that any measures utilized by a specific research community must therefore be merely conventional (Wilholt, 2009). For example, the level of statistical significance that determines the degree of confirmation needed to accept a hypothesis is a methodological convention of a research community. This level can, in principle, vary between scientific disciplines and contexts of investigation. However, even though these standards are merely conventional, they nevertheless serve an important epistemic function. Without such common methodological standards, a research community could not coordinate their research activities in a proper manner. Methodological conventions are needed to establish mutual trust in the results of research between the members of a scientific community. To see this, consider for example, a research community that employs various levels of statistical significance (say 0.05, 0.07, and 0.09), allowing hypotheses to be accepted or rejected depending on the chosen level of significance. This ambiguity would lead to confusion about what statistical significance means and which studies should be accepted as making valid claims. It would thus undermine the reliability of research results, and therefore also the coordination of collective processes of knowledge production.

We can now attend to an important difference between Reutlinger's version of EA and Wilholt's version of SEA. Wilholt's account accepts the value-ladenness of scientific inquiry and so centers on the issue of what degree of evidential confirmation C is needed to accept a hypothesis H in a given context. In contrast, Reutlinger's account focuses on the question of whether the evidence available suffices for the researcher to accept hypothesis H. For Wilholt, therefore, it is not enough to show for a given hypothesis H that P(H|E, K) > P(H|K). It is more important, on this SEA account, to show that the probability of H given a set of data E and relevant background knowledge K is sufficiently high to accept H, that is, to show that it exceeds a certain threshold of evidential confirmation.^15^ More formally:

P(H|E, K) > C > P(H|K)

The crucial question for Wilholt's account, therefore, is how to determine the exact threshold level of confirmation C such that P(H|E, K) justifies believing H given the available evidence and background information. This threshold can only be established conventionally.^16^ It is therefore impossible to evaluate the epistemic merits of accepting or rejecting a hypothesis solely by assessing all of the local evidence using Bayesian confirmation theory.

Reutlinger (2020b) highlights an obvious problem with the role of such conventional thresholds in hypothesis confirmation. Epistemic wrongness can be conventionally defined in terms of undermining collective epistemic practices that establish a specific threshold C, but it remains unclear why infringing such a convention would be *epistemically unjustified*, for it might be that the chosen level for C is epistemically inadequate. Consider, for example, the Bisphenol A case in which, according to Wilholt's analysis, the epistemic shortcoming consisted in the researcher's infringement of the methodological convention not to use CD(SD) rats in experiments to determine the carcinogenic effects of Bisphenol A. Now, one might say that the reason for this convention was that evidence gathered using CD(SD) rats does not raise P(H|E,K) above C. However, if not using the rats was merely a convention grounded in practical rather than epistemic considerations, it seems difficult to argue that the researchers made an *epistemic* mistake by using the rats.

If methodological standards are merely conventional, there is no *epistemic* reason to believe that one standard is more apt than another. In Wilholt's framework, methodological conventions are chosen because of their functionality for coordinating collective practices, not because they provide epistemic justification in terms of evidential confirmation (Wilholt, 2013). How important is this critique of Wilholt's SEA? In offering a social–epistemological account, Wilholt is not committed to individualistic conceptions of knowledge and justification. If one conceives of scientific knowledge as something produced by a collective, epistemic justification cannot reside in the reasons and evidence of any individual researcher. It must instead reside in the way a scientific community organizes the social practice of confirming and refuting hypotheses. This, however, raises the question on how to determine whether these social practices are epistemically adequate and successful in producing reliable results.

In defending his social epistemological account, Wilholt emphasizes the role of the division of cognitive labor in science (Wilholt, 2013, 2016). If science can only be epistemically successful as a collective endeavor, the criterion for assessing the aptness of conventional methodological standards must be the capacity of these standards to enable collaboration between scientists and mutual epistemic trust in their ability and willingness to report reliable results. Trust and reliability can thus themselves be considered epistemic criteria insofar as they are important to the sharing of research results and hence to the effectiveness of the division of cognitive labor and the collective search for truth. While this argument does not directly explain why it is *epistemically unjustified* for an individual researcher to infringe conventional methodological standards, it shows that such standards have a crucial epistemic function and that failure to abide by them can undermine the collaborative production of scientific knowledge. In SEAs, questions about epistemic justification must be answered with reference to the degree to which the relevant social practices are functional for bringing about beliefs that are appropriately sensitive to the relevant evidence.

We can now consider how these two paradigmatic accounts of epistemic wrongness would treat the anti-arrhythmic drug case, as described by Holman. The *Challenging the Evidential Account* section and *The Problem of the Target Level* section will discuss problems with EAs. The *Challenging the Social Epistemological Account* section formulates a challenge to SEAs and discusses a possible response.

## Challenging the Evidential Account

In this section, I will argue that EAs are ill suited to account for the influence of the pharmaceutical industry in AAC because of their focus on evidential confirmation. EAs ask whether individual researchers were justified in making a claim based on the evidence available to them. What does this mean for AAC? Were the researchers involved in the pharmaceutical industry's studies justified in believing that anti-arrhythmic drugs not only suppressed VEBs but were also therapeutically efficient? I think that they were, given the officially held and widely disseminated background belief that the VEB hypothesis was true and the preliminary evidence from *in vitro* and animal studies, which supported the existence of a causal mechanism linking anti-arrhythmic drugs to VEB suppression. The mechanistic evidence E combined with the background belief B (that VEB suppression prevents heart failure and death) to provide stronger support for the hypothesis H that the anti-arrhythmic drugs were therapeutically efficient than was given by the background belief B alone. So P(H|E, B) > P(H|B) holds.^17^ It also seems hard to argue that the researchers violated the principle of complete local evidence. Of course, there probably were studies available to them that provided counter-evidence to the VEB suppression hypothesis. However, as these studies were far outnumbered by publications suggesting the opposite, it seems that individual researchers cannot be accused of endorsing a hypothesis contrary to considerable defeating evidence. Even if the counter-evidence was fairly considered by the researchers, they were—according to the EA—epistemically justified in drawing the conclusion that anti-arrhythmic drugs are therapeutically effective.

A natural response to this argument is to argue that every epistemic agent—and scientists in particular—has a duty to question all the background assumptions of their claims. In AAC, this would have involved questioning the plausibility of the endpoint, the reliability of the expert panel that issued it as a standard, and the mainstream opinion in the cardiology community that the VEB suppression hypothesis was true. Had researchers taken into consideration evidence about the conditions under which the decision for the endpoint was taken, they would not have been justified in accepting the background belief B that the VEB suppression hypothesis was true and that VEB suppression represented a suitable endpoint for determining the therapeutic effects of the drugs.

However, this argument is more epistemically demanding than EAs, or at least than Reutlinger's, which I here treat as paradigmatic of EAs. The principle of complete local evidence only requires assessment of the *available local* evidence (Reutlinger, 2020b). The evidence required by this argument would neither be local nor, to a large extent, available because the debates in the expert panel were not transparent to the ordinary scientist, let alone to medical practitioners or patients. Moreover, the objection requires that individual researchers be more independent from the knowledge of other scientists than is plausible and have implausibly extensive abilities to double-check every premise of their argument. Scientific research in complex areas such as medicine involves a division of cognitive labor, which, as many have recognized, requires that researchers can mutually rely on each other for the truth of their reported research results, at least to some extent. This is not to say, of course, that researchers need not or should not check whether they can reproduce each other's results. However, this is often unnecessary (e.g., when a third party has already done it) or irrelevant (as when one's conclusions do not conflict with background knowledge). While a healthy skepticism is surely helpful to the scientific endeavor, scientists must make choices about when it is appropriate to adopt a skeptical stance. Limited expertise and lack of time are simple pragmatic reasons for limiting skepticism.

One problem with EAs, therefore, seems to be that they do not take into account the social context that helps determine confirmation relationships between hypothesis H, evidence E, and alleged background knowledge K. EAs do not require researchers to check for suspiciously skewed distributions of studies providing either confirming or defeating evidence or to question the genesis of the background knowledge underlying (mainstream) work in their field. EA also lacks the means to inquire into these social conditions because it does not involve epistemic principles that work at the collective level. It does not include any rules about how the pursuit of scientific consensus should be organized so that epistemic goals can be met, and it says nothing about the distribution of true and false beliefs within a community. EA accounts are thus of limited use as tools to analyze cases of sponsorship bias. As this analysis of AAC shows, researchers can make false claims and contribute to biased research without being wrong about evidential confirmation or misunderstanding confirmation relationships altogether.

## The Problem of the Target Level

So far, my argument against EA has focused on the claims of the researchers involved in the trials that used VEB suppression as an endpoint. This focus may, however, make the argument against EA too easy because one could object that the researchers involved in these studies are not the relevant target of its analysis. Perhaps the epistemically problematic claim in AAC was the expert panel's claim that VEB suppression is a valid means of predicting the long-term survival of patients. This is the claim that should be regarded as unjustified by the standards of EA because the evidence concerning the connection between VEB suppression and heart failure was, in fact, inconclusive and therefore unsuitable to confirm or falsify the hypothesis.^18^ It was this unjustified claim that led to the methodologically flawless but erroneous research by the individual scientists.

This initially appears to be a more serious objection to my argument. I will show, however, that this response relies on a misunderstanding of the applicability of confirmation theory to this panel's decision. If one takes these constraints on its application into account, one sees that the determination of the study endpoint by the expert panel must be analyzed in ethical as well as epistemic terms, which goes beyond the scope of EA.

The objection that, for EA, the expert panel's claim is the relevant target for understanding the epistemic wrongness in AAC implies that the hypothesis “VEB suppression is a reliable indicator for therapeutic effectiveness” (H_1_) was not supported by the evidence. We do have good reasons to believe that this was the case. First, as Howick (2011) reports, when the expert panel met, studies about the supposed causal mechanism linking VEB suppression and patient survival were ambiguous. In the absence of conclusive evidence, the experts were unjustified in endorsing H_1_; they should have suspended their judgment because P(H_1_|E, K) was not, in fact, (significantly) larger than P(H_1_|K). On this view, the expert panel failed because it did not base its endorsement of H_1_ on conclusive evidence. Second, the panel did not properly acknowledge views opposing the VEB suppression hypothesis and thus did not consider potentially defeating evidence. If the panel had complied with the principle of complete local evidence, P(H_1_|E, K) would probably have actually been smaller than P(H_1_|K), such that the rational response would have been to hold that H_1_ was false.

A proponent of EA can therefore claim that the epistemic wrongness of AAC consisted in the expert panel making a claim that was not supported by the available local evidence. However, this line of reasoning presupposes that it is possible to distinguish between two different wrongs involved in this case: the epistemic wrong involved in the panel's erroneous claim and the ethical wrong of the panel's dubious evaluation of the inductive risks associated with H_1_. This presupposition is false. I will argue that it is not possible to clearly distinguish between these two wrongs in AAC and thus that the above explanation of the EA account cannot withstand critical scrutiny. More precisely, my argument is that one can commit the epistemic wrong without committing the ethical wrong, which EA rightly acknowledges, but that one cannot commit the ethical wrong without also committing the epistemic wrong, which EA does not sufficiently acknowledge.

I shall first explain why AAC involved an ethical as well as an epistemic wrong. It is ethically wrong to prefer the hypothesis “VEB suppression is a reliable indicator for the therapeutic effectiveness of anti-arrhythmic drugs” (H_1_) over the competing hypothesis “an increased patient survival rate is a reliable indicator for the therapeutic effectiveness of anti-arrhythmic drugs” (H_2_). Such a preference is unethical because the primary aim of producing the drug should be to heal or at least to improve the health of patients after heart attacks. Preferring H_1_ over H_2_ would not be the optimal choice by this metric even if H_1_ was true. Even if the VEB suppression hypothesis was true and would guarantee patient survival, one could still not rule out possible further downstream effects on patient health. To optimally determine the potential risk of a drug, it would, in any case, have been better to choose an endpoint as far downstream as possible, which would be the death of the patient. Therefore, on the assumption that the experts on the panel were committed to improving patient health, their decision to choose VEB suppression as a general standard was not only an epistemic but also an ethical wrong.

One might resist the claim that the panel's decision constituted an ethical wrong by pointing out that death would not have been the optimal endpoint from the perspective of all involved. Such a choice would, for example, have significantly prolonged the study and thus delayed the drugs' availability. This would have delayed the treatment of patients struggling with heart disease, leading some to die prematurely. Choosing death as an endpoint would also not have helped settle academic disputes about the physiological mechanism underlying the effects of anti-arrhythmia drugs. As everyone eventually dies, death is the most unspecific endpoint possible if one is interested in the causal physiological mechanism of the drug. The expert panel thus faced a difficult trade-off between these different interests, and in choosing VEB suppression as a suitable endpoint, they granted lower priority to the interests of future patients than they should have. Setting endpoints for clinical trials is always a trade-off between different values and interests, but very strong arguments are needed to justify giving a relatively lower priority to the *prima facie* duty of benefitting the long-term health and survival of study participants and prospective patients. The panel did not seem to offer or consider any such arguments. It is plausible, therefore, that, even though the panel had to weigh competing interests, it committed an ethical wrong by, at the least, failing to provide an ethical justification for its decision to favor VEB suppression as a clinical endpoint.

Having established that the expert panel committed an ethical and an epistemic wrong in AAC, I shall now argue that EA does not appropriately account for the dependence relation between these wrongs. This leads EA to blur the distinction between error and bias and to unduly ignore the influence of relevant non-epistemic factors on epistemic processes. I will now show that the epistemic wrong of accepting H_1_ despite inconclusive evidence depends on the ethical wrong of preferring H_1_ over H_2_.

It is certainly logically possible to commit the epistemic wrong without committing the ethical wrong. An expert panel might wrongly conclude that the available local evidence favors H_1_ and yet judge that establishing H_1_ as an endpoint in a clinical trial is ethically unjustified. One can consistently endorse the truth of H_1_ and deny that H_1_ is an ethically justifiable endpoint. However, it is impossible to commit the ethical wrong without also committing the epistemic wrong. An expert panel cannot consistently hold that it would be ethically acceptable to define H_1_ as the study endpoint while also holding that H_1_ is wrong. Anyone committing the ethical wrong necessarily also commits the epistemic wrong. One simply cannot consistently opt for a study endpoint that one believes has nothing to do with the causal effects of the drug. EA cannot properly account for this relationship between the ethical wrong and the epistemic wrong.

EA describes AAC's epistemic problem solely with respect to the fallacious endorsement of H_1_, and thus abstracts away from the social conditions that bring this mistake about, including the ethical wrong. EA does not take into account that the practical interests that led to the ethical wrong also implied the epistemic wrong. EA can explain *why* the expert panel's conclusion was wrong, but it cannot account for *how* the social circumstances contributed to the panel reaching this wrong conclusion. EA thus construes AAC as a case of collective cognitive error rather than of genuine bias. By disconnecting the cognitive aspect of bias from the non-epistemic, social aspects that cause the error, EA fails to distinguish between error as a mere epistemic failure and bias as an epistemic failure caused by non-epistemic motives.

This becomes more obvious when we consider the epistemic failure in relation to the expert panel's task of evaluating the competing hypotheses H_1_ and H_2_ in order to determine the appropriate endpoint for the study. As the case is described, the expert panel was not epistemically justified in accepting either H_1_ or H_2_. In the case of H_2_, this is because there was no known biochemical mechanism leading from the use of the drug to the survival of the patient. Patient survival thus could not have indicated any therapeutic effect, let alone a specific causal effect of the anti-arrhythmic drugs. So, with respect to H_2_, the expert panel should have suspended judgment. As there was insufficient evidence for accepting either H_1_ or H_2_, the expert panel had no epistemic reason to prefer either hypothesis. Given that the panel's task was to decide which of the two hypotheses was better supported by the evidence, by the standards of EA, it should not have endorsed either of them. It should instead have concluded that the evidence was inconclusive and that more research was needed.

If this analysis is correct, proponents of EA will struggle to explain how the epistemic error could have occurred without accepting that non-epistemic reasons were decisive, such as the pressure to reach a decision. The epistemic wrong certainly consisted in falsely asserting that H_1_ was true, but given that both options available to the panel were epistemically problematic, the only possible explanation for their decision is their preference for H_1_.

Seen this way, the expert panel's task was not to determine which of the two competing hypotheses was better supported by the evidence, but what kind of standard for epistemic justification was acceptable in this case. This is an evaluative question that cannot be answered by evidential considerations alone.

It is significant that EA is neutral on the question of whether science should be conceived of as value-free. Reutlinger regards this neutrality as an advantage (Reutlinger, 2020b). The above discussion, however, shows that EA is of limited use in cases like AAC because the expert panel was making a decision about the proper standards for epistemic justification. Such decisions involve an assessment of the ethical consequences of choosing one standard over the other, and hence involve value judgments. So, if proponents of EA wish to insist that the relevant instance of epistemic wrongness is to be located on the level of the expert panel, they cannot maintain that an account of epistemic wrongness can properly ignore the role of values in science and focus only on narrower evidential concerns. In sum, EA lacks the resources to explain AAC as a case of epistemic wrongness.

## Challenging the Social Epistemological Account

In order to reach a fully considered decision between the two proposed analyses of sponsorship bias, it is necessary to also consider how the social epistemological account treats AAC. I will argue that AAC also poses a challenge to SEA, but I will also argue that SEA has better resources than EA to respond to this challenge.

In AAC, the expert panel established a corrupted methodological standard. Therefore, it seems that one cannot explain the epistemic wrongness of the case in terms of individual researchers infringing that standard. We still might want to say that evidence produced by the pharmaceutical industry was biased, as it was based on the corrupted methodological standard. However, we cannot make this claim on the grounds required by SEA, which invoke the epistemic practices of the whole research community. The pharmaceutical industry's research into the effectiveness of the drug was conducted on the premise that the VEB hypothesis was true and thus was perfectly in line with the conventional standard of the research community. Therefore, this research cannot be criticized for infringing a conventional methodological standard. Rather, the work of critical researchers who challenged the VEB suppression hypothesis would have to be accused of this infringement.

However, a proponent of SEA might mount a similar response to the proponent of EA and argue that the methodological standard used by the researchers is not the relevant target of epistemic critique. This standard was the result of an infringement of more general standards of scientific discourse by the expert panel and the FDA. One could argue, for example, that the expert panel in AAC infringed the rule that in, an open scientific discourse, all positions should be heard and all relevant evidence considered. Epistemic wrongness might thus still be explained as an infringement of a conventional rule. Just as an EA proponent might want to claim that the panel's decision was not properly based on complete local evidence, a proponent of SEA might want to argue that rules of building a valid scientific consensus were infringed by (some of) the experts on the panel.

From a social epistemological perspective, there are good reasons to conclude that the expert panel's decision-making process infringed the standards of an epistemically fruitful scientific discourse. Epistemologists such as Longino (1990) and Kitcher (2001) have long argued that a plurality of perspectives and a critical and open discourse are preconditions for successful scientific inquiry. From the perspective of theoretical frameworks that emphasize the collective nature of scientific knowledge, it can plausibly be argued that the rules governing these collective practices should establish these conditions in order to enable reliable knowledge production. However, it is highly doubtful that these rules should themselves be regarded as merely conventional. Such rules are valid not simply because they are conventional but because they are grounded in the epistemological principle that a proposition is more likely to be true if it can be independently confirmed from multiple perspectives. Whether a proposition can be confirmed in this way, however, is not simply a question of actually reaching an agreement, but of what the different parties deliberating about the issue actually have reason to believe. The development of collective knowledge through discourse therefore has a rational basis. From this perspective, proponents of SEA do seem to have the resources to explain what went epistemically wrong in EA's analysis of the expert panel.^19^

One might wonder whether one could make the same point from the perspective of Wilholt's specific SEA, which I introduced as a paradigmatic of the approach. Wilholt's account seems to differ from those of Longino and Kitcher because it conceives of methodological standards as somehow creating the conditions under which scientific inquiry can flourish, rather than as grounded in a foundational epistemic principle such as the diversity of perspectives. Conventional standards are epistemically relevant for Wilholt because they enable scientific inquiry as a collective endeavor. It should, however, also be possible to conceive of the failure of the expert panel as an infringement of (higher order) conventional standards from the perspective of Wilholt's account. The scientific community must be able to rely on expert panels to determine methodological standards in a way that ensures that research aligns with contextually relevant non-epistemic values. In AAC, these values would include, most relevantly, the value of promoting public health rather than private profit. The expert panel should have chosen a stricter standard than VEB suppression in order to be worthy of the trust of the broader scientific community. This analysis assumes that methodological standards should be representative of the shared values of the members of the scientific community. The irony is that a conventional standard can only enable the epistemic trust that Wilholt's account demands if it is representative of the shared values of the research community. From the perspective of a social epistemological account like Wilholt's, the expert panel in AAC can be seen to have disregarded the relevant values of the scientific community. It thereby not only implemented a dysfunctional standard that did not enable epistemic trust, but also infringed the (implicit) norm of finding a standard that was representative of the values of the research community, and not the pharmaceutical industry.

If we accept this analysis, then we can see that social epistemological accounts provide a more plausible analysis than evidential accounts of the kind of manufactured certainty seen in AAC. I conclude that, insofar as AAC represents a case of sponsorship bias, SEA has more explanatory power. This suggests that it is more fruitful to assess the epistemic wrongness of sponsorship bias from a social epistemological rather than an individualist perspective. Focusing only on relationships of evidential support not only neglects the causal influence on research practices of the preferences of various stakeholders and how they shape the evaluation of evidential support relationships but also fails to account for the role of values and decision making in scientific research. As AAC shows, the latter is crucial, at least for some paradigmatic cases of sponsorship bias.

## Conclusion

This paper compared two recent accounts of the epistemic wrongness of sponsorship bias (SB): the evidential account (EA) and the social epistemological account (SEA). The advantages and disadvantages of these accounts were illuminated by applying them to a paradigmatic case of sponsorship bias. This case can be interpreted as one of manufactured certainty, in which the financial interests of stakeholders contributed to the establishing of epistemically inadequate methodological standards.

Evidential accounts give a convincing account of what goes epistemically wrong in many cases of sponsorship bias and identify the fundamental epistemic flaw as involving the making of assertions that are not backed up by the available local evidence or that misunderstand evidential support relations. However, evidential accounts struggle to explain how these epistemic flaws are produced by the concrete epistemic practices of knowledge-producing community. As a result, they struggle to properly distinguish between bias and error, and also to account for cases such as AAC, which involve infringements of the normative structure of scientific research. Social epistemological accounts, on the other hand, can quite easily explain how practices lead to instances of bias because they explain the epistemic wrongness of bias in terms of breaking the conventions of scientific practice. However, as a result of their emphasis on practices and conventions, SEAs in turn face the problem of providing an epistemological basis for evaluating the infringement of merely conventional standards. I have argued that this problem can be resolved by supplementing the conventional view of epistemic wrongness with a robust social epistemology that, like Wilholt's view, explains the epistemic significance of conventions through their relevance to collective processes of knowledge generation. More importantly, however, SEAs, unlike EAs, also have the conceptual resources to explain cases of sponsorship bias such as AAC because their focus on collective practices facilitates analysis of decision-making processes that are responsive to values as well as to evidence. These cases suggest that an alleged advantage of EAs, that they can remain neutral regarding the value-ladenness of science, is actually a disadvantage. The inability of EA to properly distinguish bias and error is an expression of exactly this disadvantage. Approaches like SEA, that link epistemological concerns with concerns about the role of social and ethical values in science, are thus more useful than EA for research into sponsorship bias.

## Data Availability Statement

The original contributions presented in the study are included in the article/supplementary material, further inquiries can be directed to the corresponding author.

## Author Contributions

JL declares that the complete manuscript is product his own research and writing.

## Conflict of Interest

The author declares that the research was conducted in the absence of any commercial or financial relationships that could be construed as a potential conflict of interest.

## Footnotes

^1^In focusing on sponsorship bias, I do not wish to insinuate that all interactions between public research and private sponsors are necessarily epistemically detrimental or ethically dubious. Private sponsorship can have advantages: In applied research, there are various examples of collaboration between publicly and privately funded researchers producing epistemically and socially valuable results. Moreover, private funding sometimes enables research that would not otherwise be possible due to a lack of public funding (Wilholt, 2006). Collaboration between industries and public research institutions can also sometimes accelerate and intensify research, as shown by the recent development of several vaccines against COVID-19 infection through the efforts of researchers in competing biotech companies and public universities. While these positive effects of private research funding are frequently mentioned in the literature (Adam et al., 2006; Carrier, 2008; Holman and Elliott, 2018), thorough philosophical and sociological investigation is needed to determine the exact conditions under which competition and collaboration between publicly and privately funded research have positive or negative epistemic effects.

^2^For an overview and various examples of these biases, see Wilholt (2009) and Holman and Elliott (2018).

^3^In this paper, I am only concerned with a form of bias that involves some kind of practical interest. In the literature, the term “bias” is sometimes used more broadly to also encompass cases of false reasoning and unjustified belief formation, irrespective of whether or not they originate in a conscious practical interest. In the current literature, “bias” is often conceived of as an implicit prejudice against a (social) object (Beeghly and Madva, 2020). Biased reasoning is thought to result from the influence of various social, cultural, or economic factors on human cognition. For examples of the variety of uses of the term, see Goldman (1999), Resnik (2000).

^4^I refer to science as value-free only with respect to non-epistemic, *viz*., social, political, or moral values. I am not here interested in discussions of the role of epistemic or cognitive value judgments in theory choice. See, for instance, Kuhn (1977/2000) and Douglas (2013) on this point.

^5^The argument from inductive risk is just one example that shows that value-free science is, at best, an unreachable ideal. There are other arguments for this conclusion and in much of the sociology of science literature, the impossibility of value-free science is even taken for granted (Barnes et al., 1996). Adjudicating this complex issue is, however, far beyond the scope of this paper.

^6^Though endorsed by many, the claim that scientific practice necessarily involves non-epistemic value judgments is contested. There is, in fact, a particular debate about the argument from inductive risk (Betz, 2013, 2017; Melo-Martín, 2016).

^7^This is, however, not to say that one cannot justify the validity of epistemic and non-epistemic values by assessing their effects on epistemic practices. For instance, one can validate (non-)epistemic values that shape scientific practices by their long-term empirical success, i.e., the involvement of (non-)epistemic values in a scientific practice is justified insofar as they contribute to the overall success of the practice to produce empirical knowledge. Ohnesorge (2020) has recently made a similar point. I thank an anonymous reviewer for reminding me of this.

^8^Holman is not concerned with sponsorship bias in the cited paper, but rather with the preconditions of a practically relevant medical epistemology. Accordingly, he uses both versions of the case to illustrate how the traditional, individualist epistemology that underlies Howick's criticism of mechanistic evidence fails to account for the financial, social, and political interests involved in the determination of epistemic standards in medicine.

^9^At least this seems a plausible reading. One might, however, object that researchers did not actually claim that VEB suppression was sufficient evidence for therapeutic effectiveness, but that this claim was merely an implication of the regulations issued by the FDA to approve the respective drugs. We will return to this issue in *The Problem of the Target Level* section.

^10^For a comparison see, for example, the analysis by Oreskes and Conway (2010).

^11^There are also views in the philosophy of science that deny the possibility of evidential confirmation altogether (Popper, 1959/2008). However, falsificationism gains much of its plausibility from its argument against an absolute understanding of confirmation, as opposed to a probabilistic understanding. Current theories of evidential confirmation, however, are invariably probabilistic.

^12^The original description of the case can be found in Proctor (2012).

^13^Blaming someone for an error only seems justified if one supposes that the person should have known the norms that she violated when making the error. What someone in a certain situation could or should have known, however, depends on social norms about what we can reasonably expect each other to know.

^14^I believe that this coupling of bias and error represents a major problem with Reutlinger's account. There are important differences between our ordinary concepts of bias and error that cannot be accounted for in purely epistemic terms. Bias implies that violations of valid epistemic principles are brought about in a way that involves *specific* and wrongful epistemic practices. For example, one can make an error due to negligence, inattentiveness, or bad luck, but one's reasoning is not rendered biased by such cognitive failures alone. The term bias refers to *structural conditions* (cognitive or social) that systematically influence the epistemic practices that justify one's beliefs. An error is just the *result* of processes that are influenced by these cognitive or social conditions. Reutlinger's account seems to blur the distinction between bias and error because his focus on epistemic wrongness leaves out the conditions of error formation that are decisive for understanding bias. Insofar as these structural factors are decisive, his evidential account of epistemic wrongness does not sufficiently discriminate between error and bias as two subspecies of flawed research and, hence, does not have the resources to fully explain the phenomenon of sponsorship bias. Reutlinger might respond that his primary goal was only to deliver an account of epistemic wrongness, which might later be supplemented with more concrete descriptions of the mechanisms that make the epistemic wrong more likely to occur in cases of sponsorship bias. However, while describing such mechanisms would surely be helpful, it remains unclear how such a description relates to the occurrence of error. What needs to be shown is how, for example, conflicting interests or financial incentives make the occurrence of the described error more likely than in cases in which these influences are absent.

^15^In this respect, Wilholt's account is reminiscent of Lockean approaches to the rationality of belief revision [Foley (1992)]. According to the so-called Lockean thesis, an epistemic agent who assigns credence to propositions in proportion to the available evidence is required to believe all and only those propositions to which she assigns sufficiently high credence, *viz*., credence above some threshold level t (Shear and Fitelson, 2019). I am grateful to an anonymous reviewer for making me aware of this point.

^16^It should be obvious that this account requires, at the minimum, that C > 0.5. However, how much larger than 0.5 the threshold must be cannot be determined by the available evidence.

^17^I use the variable B instead of K because obviously the background belief is in fact false and, hence, does not amount to background knowledge.

^18^This argument echoes Howick's argument for preferring statistical over mechanistic evidence in therapeutic decision making. Pathophysiological mechanisms are often unknown or too complex to allow for definite predictions (Howick, 2011). Consequently, it is too risky to rely on them when there is a lot at stake. As Howick interprets the case, the failure of the experts was exactly this—they relied on “low quality” mechanistic evidence about the connection between VEB suppression and death, when statistical evidence would have been more appropriate.

^19^SEA might also identify other epistemic errors in AAC. For instance, one might also argue from a social epistemological perspective that the marketing strategies applied by the pharmaceutical industry infringed standards of transparency or that the one-sided funding of friendly research violated the requirement to give equal consideration to different perspectives.

## References

[B1] Adam M. Carrier M. Wilholt T. (2006). How to serve the customer and still be truthful: methodological characteristics of applied research. Sci. Public Policy 33, 435–444. 10.3152/147154306781778849

[B2] Barnes B. Bloor D. Henry J. (1996). Scientific Knowledge: A Sociological Analysis. Chicago, IL: University of Chicago Press.

[B3] Beeghly E. Madva A. (eds.) . (2020). An Introduction to Implicit Bias: Knowledge, Justice, and the Social Mind. New York, NY: Routledge.

[B4] Betz G. (2013). In defence of the value free ideal. Eur. J. Philos. Sci. 3, 207–220. 10.1007/s13194-012-0062-x

[B5] Betz G. (2017). Why the argument from inductive risk doesn't justify incorportaing non-epistemic values in scientific reasoning, in Current Controversies in Values and Science, eds Elliott K. C. Steel D. (New York, NY, London: Routledge), 94–110.

[B6] Biddle J. B. Leuschner A. (2015). Climate skepticism and the manufacture of doubt: can dissent in science be epistemically detrimental? Eur. J. Philos. Sci. 5, 261–278. 10.1007/s13194-014-0101-x

[B7] Brown J. R. (2008). The community of science®, in The Challenge of the Social and the Pressure of Practice: Science and Values Revisited, eds Howard D. Kourany J. A. Carrier M. (Pittsburgh, PA: University of Pittsburgh Press), 189–216.

[B8] Carrier M. (2008). Science in the grip of the economy: on the epistemic impact of the commercialization of research, in The Challenge of the Social and the Pressure of Practice: Science and Values Revisited, eds Howard D. Kourany J. A. Carrier M. (Pittsburgh, PA: University of Pittsburgh Press), 217–234.

[B9] Carrier M. (2013). Values and objectivity in science: value-ladenness, pluralism, and the epistemic attitude. Sci. Educ. 22, 2547–2568. 10.1007/s11191-012-9481-5

[B10] Douglas H. E. (2000). Inductive risk and values in science. Philos. Sci. 67, 559–579. 10.1086/392855

[B11] Douglas H. E. (2007). Rejecting the ideal of value-free science, in Value-Free Science? Ideals and illusions, eds Wylie A. Kincaid H. Dupré J. (Oxford, New York, NY: Oxford University Press), 120–139.

[B12] Douglas H. E. (2009). Science, Policy, and the Value-Free Ideal. Pittsburgh, PA: University of Pittsburgh Press.

[B13] Douglas H. E. (2013). The value of cognitive values. Philos. Sci. 80, 796–806. 10.1086/673716

[B14] Earman J. (1992). Bayes or Bust? A Critical Examination of Bayesian Confirmation Theory. Cambridge, MA: MIT Press.

[B15] Fernandez Pinto M. (2017). To know or better not to: agnothology and the social construction of ignorance in commercially driven research. Sci. Technol. Stud. 30, 53–72. 10.23987/sts.61030

[B16] Foley R. (1992). The epistemology of belief and the epistemology of degrees of belief. Am. Philos. Q. 29, 111–124.

[B17] Goldman A. I. (1999). Knowledge in a Social World. Oxford, New York, NY: Clarendon Press.

[B18] Hempel C. G. (1965). Science and human values, in Aspects of Scientific Explanation and Other Essays in the Philosophy of Science, ed Spiller R.E. (New York, NY, London: Free Press; Collier Macmillan), 81–96.

[B19] Holman B. (2019). Philosophers on drugs. Synthese 196, 4363–4390. 10.1007/s11229-017-1642-2

[B20] Holman B. Bruner J. (2015). The problem of intransigently biased agents. Philos. Sci. 82, 956–968. 10.1086/683344

[B21] Holman B. Bruner J. (2017). Experimentation by Industrial Selection. Philos. Sci. 84, 1008–1019. 10.1086/694037

[B22] Holman B. Elliott K. C. (2018). The promise and perils of industry-funded science. Philos. Compass 13:e12544. 10.1111/phc3.12544

[B23] Howick J. (2011). The Philosophy of Evidence-Based Medicine. Chichester: Wiley-Blackwell BMJ Books.

[B24] Hume D. (1748/2009). An Enquiry Concerning Human Understanding: A Critical Edition. Oxford: Clarendon Press.

[B25] Kitcher P. (2001). Science, Truth, and Democracy. New York, NY: Oxford University Press.

[B26] Kuhn T. S. (1977/2000). Objectivity, value judgement, and theory choice, in The Essential Tension: Selected Studies in Scientific Tradition and Change, ed Kuhn T. (Chicago, IL: University of Chicago Press), 320–339.

[B27] Lexchin J. Bero L. A. Djulbegovic B. Clark O. (2003). Pharmaceutical industry sponsorship and research outcome and quality: systematic review. BMJ 326, 1167–1170. 10.1136/bmj.326.7400.1167 12775614 PMC156458

[B28] Longino H. E. (1990). Science as Social Knowledge: Values and Objectivity in Scientific Inquiry. Princeton, NJ: Princeton University Press.

[B29] Lown B. (1979). Sudden cardiac death: the major challenge confronting contemporary cardiology. Am. J. Cardiol. 43, 313–328. 10.1016/S0002-9149(79)80021-1 83793

[B30] Lundh A. Lexchin J. Mintzes B. Schroll J. B. Bero L. (2017). Industry sponsorship and research outcome. Cochrane Database Syst. Rev. 2:MR000033. 10.1002/14651858.MR000033.pub3 28207928 PMC8132492

[B31] Mayo D. G. (2011a). Error and the Growth of Experimental Knowledge. Chicago, IL: Univ. of Chicago Press..

[B32] Mayo D. G. (2011b). Learning from error, severe testing, and the growth of theoretical knowledge, in Error and Inference: Recent Exchanges on Experimental Reasoning, Reliability, and the Objectivity and Rationality of Science, eds Spanos A. Mayo D. G. (Cambridge: Cambridge University Press), 28–57.

[B33] Melo-Martín I. de. (2019). The commercialization of the biomedical sciences: (mis)understanding bias. Hist. Philos. Life Sci. 41:34. 10.1007/s40656-019-0274-x 31485872

[B34] Melo-Martín I. de Intemann K. (2016). The risk of using inductive risk to challenge the value-free ideal. Philos. Sci. 83, 500–520. 10.1086/687259

[B35] Ohnesorge M. (2020). The limits of conventional justification: inductive risk and industry bias beyond conventionalism. Front. Res. Metr. Anal. 5:599506. 10.3389/frma.2020.599506 33870060 PMC8025966

[B36] Oreskes N. Conway E. M. (2010). Merchants of Doubt: How a Handful of Scientists Obscured the Truth on Issues From Tobacco Smoke to Global Warming. New York, NY: Bloomsbury Press.

[B37] Popper K. R. (1959/2008). The Logic of Scientific Discovery. London: Routledge.

[B38] Proctor R. N. (2012). Golden Holocaust: Origins of the Cigarette Catastrophe and the Case for Abolition. Berkeley, CA: University of California Press.

[B39] Resnik D. B. (2000). Financial interests and research bias. Perspect. Sci. 8, 255–285. 10.1162/106361400750340497

[B40] Reutlinger A. (2020a). Strategischer Wissenschaftsskeptizismus, in Wissenschaftsreflexion: Interdisziplinäre Perspektiven auf Theorie, Praxis und Ethik der Wissenschaften, eds Jungert M. Frewer A. Mayr E. (Paderborn: Mentis), 351–370.

[B41] Reutlinger A. (2020b). What is epistemically wrong with research affected by sponsorship bias? the evidential account. Eur. J. Philos. Sci. 10:g5949. 10.1007/s13194-020-00280-2

[B42] Robinson M. D. (2019). Financializing epistemic norms in contemporary biomedical innovation. Synthese 196, 4391–4407. 10.1007/s11229-018-1704-0

[B43] Rudner R. (1953). The scientist qua scientist makes value judgments. Philos. Sci. 20, 1–6. 10.1086/287231

[B44] Shear T. Fitelson B. (2019). Two approaches to belief revision. Erkenntnis 84, 487–518. 10.1007/s10670-017-9968-1

[B45] Sismondo S. (2008). Pharmaceutical company funding and its consequences: a qualitative systematic review. Contemp. Clin. Trials 29, 109–113. 10.1016/j.cct.2007.08.001 17919992

[B46] Sprenger J. Hartmann S. (2019). Bayesian Philosophy of Science. Oxford: Oxford University Press.

[B47] vom Saal F. S. Hughes C. (2005). An extensive new literature concerning low-dose effects of bisphenol A shows the need for a new risk assessment. Environ. Health Perspect. 113, 926–933. 10.1289/ehp.7713 16079060 PMC1280330

[B48] Wilholt T. (2006). Design rules: industrial research and epistemic merit^*^. Philos. Sci. 73, 66–89. 10.1086/510175

[B49] Wilholt T. (2009). Bias and values in scientific research. Stud. Hist. Philos. Sci. Part A 40, 92–101. 10.1016/j.shpsa.2008.12.005

[B50] Wilholt T. (2013). Epistemic trust in science. Br. J. Philos. Sci. 64, 233–253. 10.1093/bjps/axs007

[B51] Wilholt T. (2016). Collaborative research, scientific communities, and the social diffusion of trustworthiness, in The Epistemic Life of Groups: Essays in the Epistemology of Collectives, eds Brady M. Fricker M. (Oxford: Oxford University Press), 218–235.

